# Pinning potential in highly performant CaKFe_4_As_4_ superconductor from DC magnetic relaxation and AC multi-frequency susceptibility studies

**DOI:** 10.1038/s41598-022-23879-2

**Published:** 2022-11-09

**Authors:** A. M. Ionescu, I. Ivan, D. N. Crisan, A. Galluzzi, M. Polichetti, S. Ishida, A. Iyo, H. Eisaki, A. Crisan

**Affiliations:** 1 National Institute of Materials Physics, 405A Atomistilor Str., 077125 Magurele, Romania; 2 Department of Physics “E.R. Caianiello”, University of Salerno, Via Giovanni Paolo II, 132 84084Fisciano, Italy; 3 Research Institute for Advanced Electronics and Photonics, National Institute of Advanced Industrial Science and Technology (AIST), 1-1-1 Umezono, Tsukuba, Ibaraki 305-8568 Japan

**Keywords:** Condensed-matter physics, Materials for devices, Materials for energy and catalysis, Materials science

## Abstract

We have investigated the pinning potential of high-quality single crystals of superconducting material CaKFe_4_As_4_ having high critical current density and very high upper critical field using both magnetization relaxation measurements and frequency-dependent AC susceptibility. Preliminary studies of the superconducting transition and of the isothermal magnetization loops confirmed the high quality of the samples, while temperature dependence of the AC susceptibility in high magnetic fields show absolutely no dependence on the cooling conditions, hence, no magnetic history. From magnetization relaxation measurements were extracted the values of the normalized pinning potential U*, which reveals a clear crossover between elastic creep and plastic creep. The extremely high values of U*, up to 1200 K around the temperature of 20 K lead to a nearly zero value of the probability of thermally-activated flux jumps at temperatures of interest for high-field applications. The values of the creep exponents in the two creep regimes resulted from the analysis of the magnetization relaxation data are in complete agreement with theoretical models. Pinning potentials were also estimated, near the critical temperature, from AC susceptibility measurements, their values being close to those resulted (at the same temperature and DC field) from the magnetization relaxation data.

## Introduction

The discovery of iron-based superconductors (IBS) La(O_1−x_Fe_x_)FeAs, (x = 0.05–0.12)^1^ with a critical temperature *T*_c_ = 26 K, with unique superconducting mechanism and potential of use in various applications led to a world-wide effort in the discovery and comprehensive studies of this new class of superconducting materials. Up to now there are literally thousands of possible chemical compositions and doping levels of various IBS, of different types of crystalline structure. Among those different types of IBSs reported in the literature, 1111-type *RE*FeAsO_1−x_ (*RE* being a rare-earth element) has the highest critical temperature of 55 K^2^. Another type, namely 11-type Fe(Se, Te), have relatively low *T*_c_ (10–20 K depending on composition), but is promising as thin film, having a high critical current density *J*_c_ in very high magnetic field (over 10^4^ A/cm^2^ in fields larger than 10 T)^3^. Lately, superconductors based on *AE*Fe_2_As_2_ (*AE* being alkali-earth metal Ca, Sr, Ba) parent compound, the so-called 122 system, became the most popular materials for both physical explorations and wire applications because of their *T*_c_ as high as 38 K^4^, very high upper critical fields µ_0_*H*_c2_ (> 70 T)^5,6^ and low anisotropies γ (< 2)^6^, attracting substantial attention in comparison with other IBSs that have been reported in the literature. Superconductivity in *AE*Fe_2_As_2_ is primarily induced by alkali metal (*A* = Na, K, Rb, Cs) substitution at *AE* sites. The structure’s crystallographic space group (*I4/mmm*) is not changed by this *A* substitution because *AE* and *A* randomly occupy crystallographically equivalent sites. Thus, (*AE*_1−x_*A*_x_)Fe_2_As_2_ (also noted as (*AE*,*A*)Fe_2_As_2_) are solid solutions between *AE*Fe_2_As_2_ and *A*Fe_2_As_2_ compounds with the same structural type. It was found that, if there is a large difference in the ionic Δ*r* radii of *AE* and *A*, such solid solutions are not possible.

More recently, a new type of IBSs has been reported^7^, having a new structure, abbreviated as *AEA*1144, namely Ca*A*Fe_4_As_4_ (*A* = K, Rb, Cs) and Sr*A*Fe_4_As_4_ (*A* = Rb, Cs). In these cases, because *A* does not mix with *AE* due to the large Δ*r*, *AEA*1144 crystallizes through alternate stacking of the *AE* and *A* layers across the Fe_2_As_2_ layer, changing the space group from *I4/mmm* to *P/4 mmm,* the compounds being superconductors with *T*_c_ values between 31 and 36 K. The most studied compound in this new type of IBS is CaKFe_4_As_4_ (CaK1144) due to its excellent superconducting properties. The fabrication of high-quality single crystals of CaK1144 by high-temperature solution growth out of FeAs flux^8^ paved the way for a large number of studies of fundamental properties of this new material. It was proved that CaK1144 is a multi-gap (multi-component) superconductor using several techniques: muon spin relaxation (µSR)^9^, inelastic neutron scattering^10^, µSR and angle-resolved photoemission spectroscopy (ARPES)^11^, London penetration depth and tunneling conductance^12^,^75^As nuclear magnetic resonance (NMR)^13^. The results of the above-mentioned experiments are consistent with a two-gap *s* + *s*− wave model. It is interesting that CaK1144 does not undergo any magnetic or structural transition^8^, unlike many other IBSs, and, having a mean free path of the carriers six times larger than the coherence length, this system is in the clean limit. More recently it was shown that the temperature dependence of the upper critical field can be well described by a two-band model in the clean limit with band-coupling parameters favoring intraband over interband interactions^14^. However, the band structure of CaK1144 is not that simple. A recent study on the anisotropy of the London penetration depth λ_L_, measured with a microwave-coplanar-resonator technique that allowed to de-convolute the anisotropic contributions λ_L,*ab*_ and λ_L,*c*_, compared the resulted anisotropy parameter γ_L_ = λ_L,*c*_/λ_L,*ab*_, and its temperature dependence with theoretical results of *ab-initio* density-functional-theory (DFT)^15^. DFT calculations show that the Fermi surface consists of five bands centered around Γ with a hole character and three bands centered around *M* with an electron character. Furthermore, the behavior of the band-resolved density-of-states (DOS) supports the choice to employ a three-component model.

From the point of view of potential applications, the behavior of the critical current density, and the structure and type of defects that act as effective pinning centres in high magnetic fields are of paramount importance. Various kinds of defect structures have been observed as conductance modulation and topographic features by scanning tunneling microscopy (STM)^16^, and effects of artificial defects (chemically-induced by Ni substitution or irradiation-induced using 3.5 MeV protons) on the intrinsic anisotropy determined by the previously-mentioned^15^ microwave-coplanar-resonator technique.

For high-density polycrystalline samples obtained via a mechanochemically assisted synthesis route, magnetic hysteresis loops and subsequent analysis showed the presence of strong point pinning centers^17^, with values of critical current density that can be further improved by manipulating the fabrication process. For high-quality CaK1144 single crystals, critical current densities have been determined from DC magnetic hysteresis loops. The most important result is that *J*_c_ for magnetic fields parallel with *ab*-planes, *J*_c//ab_, is significantly larger than *J*_c_ for fields parallel with the *c*-axis, *J*_c//c_^18–20^. Also, it was found a local maximum in *J*_c//ab_ at fields around 1 T and a non-monotonic temperature variation of *J*_c//ab_ at high fields^19,20^. Transmission electron microscopy (TEM) studies showed the presence of a high-density of planar defects consisting of nanoscale intergrowth of CaFe_2_As_2_ layers, with an average separation of 50 nm, and they were suggested as dominant pinning centers for vortices along the *ab*-plane, and contributing to the enhanced *J*_c//ab_ at around 1 T^20^. More recently, high-resolution scanning TEM coupled with electron energy loss spectroscopy demonstrated that these planar defects consist of one- or two-layer KFe_2_As_2_ step network, which is spread over the periodically ordered KFe_2_As_2_ and of CaFe_2_As_2_ monolayers, while the dark contrast regions observed via conventional STEM are local strain distributions which originate from the substitution of Ca by K in a unit cell^21^. It was found that such CaFe_2_As_2_ and KFe_2_As_2_ intergrowth can also be thicker, and they can induce different types of pinning centers. Apart from their dimensionality, pinning centers can be either δ*T*_c_-type, meaning a local change in the critical temperature, or δ*l*-type, meaning a local change in the mean free path of the carriers. From studies of AC susceptibility and dynamic relaxation^22^ it was shown that the thick CaFe_2_As_2_ intercalations (non-superconducting) act like weak Josephson junctions. In the same study it was shown that the thin CaFe_2_As_2_ intercalations are superconducting (with a lower *T*_c_) due to charge transfer from the CaKFe_4_As_4_ matrix, and they induced both types of pinning centers. In low magnetic fields both types of pinning centers are effective, while at fields higher than 3 T the δ*l* pinning centers are dominant. In the same time, monolayer KFe_2_As_2_ intercalations are normal phase, thus creating single-vortex, very strong δ*T*_c_ pinning centers.

Up until now, the strength of the various types of pinning centers mentioned above, which are responsible for the excellent properties of this new material in terms of critical current density and very steep irreversibility line (crucial for high-field applications) were studied as bulk pinning force from DC magnetic hysteresis loops (e.g., see Ref.^20^). In this work, we investigate the pinning potential of the nanoscale defects (pinning centers) in CaK1144 single crystals from magnetic relaxation measurements and frequency-dependent AC susceptibility studies.

## Experimental and method

CaKFe_4_As_4_ single crystals were synthesized via the self-flux method using FeAs, the preparation being described in detail in^19^. The single crystals used in this study are thin square discs, having the typical dimensions *a* ≈ 1.2 mm, *b* ≈ 1.2 mm and *c* ≈ 0.04 mm. Standard (zero-field cooling, ZFC) DC magnetic hysteresis curves and the magnetization relaxation were registered with a commercial *Quantum Design* Magnetic Property Measurement System (MPMS). Well below *T*_c_ and above the field for the first full vortex penetration (in increasing *H*), or at any *H* in decreasing field, the irreversible magnetic moment is identified with the measured moment *m*. AC susceptibility studies were performed using a commercial *Quantum Design* Physical Property Measurement System (PPMS), with frequencies up to 10 kHz and AC field amplitudes up to 16 Oe. In all the experiments reported here, the applied field (both DC and AC) is parallel to the *c*-axis (perpendicular to the largest plane *a*-*b*) of the single crystal.

It is now well established that the use of DC magnetic relaxation measurements can provide very useful information regarding the type of vortex creep (elastic or plastic), the pinning potential (activation energy), and the vortex creep exponent *p* in the current density *J* dependence of the pinning potential^23^. The vortex exponent has well established theoretical values for various collective (elastic) vortex creep regimes^24^: 1/7 in the single vortex collective creep domain, 3/2 or 5/2^23^ in the case of the creep of small vortex bundles, and 7/9 for large vortex bundles. In a disordered vortex phase the creep process involves plastic vortex deformations, and *p* is negative (*p* =  − 0.5 for dislocation-mediated plastic creep)^25^. The lower limit for *p* is − 1 (in the linear Anderson-Kim model^26^), whereas the upper one indicated by the elastic manifold theory in the small vortex bundle creep regime is 2.5^23^. The DC magnetic relaxation results (at relatively long time *t*) are well described in terms of thermally activated flux creep^23^. After the application of a DC external magnetic field *H*, with the parameterization of the pinning potential *U* introduced in Ref.^27^ and the general vortex creep relation^28^, the *U*[*J*(*t*), *T*] dependence is expressed as

1 $$U\left[ {J\left( t \right),T} \right] \, = \, \left( {U_{{\text{c}}} /p} \right)\left[ {\left( {J_{{{\text{c}}0}} /J} \right)^{p} {-}{ 1}} \right] \, = T{\text{ln}}\left( {t/t_{0} } \right),$$

where *U*_c_(*T*) is a characteristic pinning energy, *J*_c0_(*T*) is the creep-free critical current density, and *t*_0_ is the macroscopic time scale for creep (~ 10^−6^– 1 s), varying weakly with *J*(*t*)^23^. As mentioned above, the vortex creep exponent *p* (mainly depending on the *J*/*J*_c0_ range and *H*) is positive in the case of a collective (elastic) vortex creep regime, and negative for plastic creep regime. The fit of the magnetization relaxation curves *m*_irr_(*t*) ∝ *J*(*t*) using Eq. (1) implies four parameters, and may lead to *p* values well outside the theoretically accepted interval, especially when the relaxation at short *t* is included, due to the uncertainty related to the initial *m*_irr_ in the vortex creep process. The analysis is simplified by determining a normalized relaxation rate *S* =  − *d*ln(|*m*_irr_|)/*d*ln(*t*) =  − *d*ln(*J*)/*d*ln(*t*) and the corresponding normalized pinning potential U* = *T*/*S*^29^. In the low-*T* domain, where the temperature variation of the pinning potential is weak, the main role of the thermal energy is to change the probed *J*/*J*_c0_ interval^30^, and, consequently, one can take *J* or *T* as an explicit variable in Eq. (1). At constant *H* and *T*, when the overall *J* relaxation is not pronounced, *p* and *t*_0_ can be considered constant (with a good approximation), leading to

2 $$U^{*}\left( J \right) \, = U_{{\text{c}}} \left( {J_{{{\text{c}}0}} /J} \right)^{p} = U_{{\text{c}}} \left( {\left| {m_{0} } \right|/|m_{{{\text{irr}}}} |} \right)^{p} .$$

With the above relations, one obtains U* = *U*_c_ + *pT*ln(*t*/*t*_0_). For a fixed, moderate, window time for averaging *t*_w_, ln(*t*/*t*_0_) ~ ln(*t*_w_/*t*_0_) ~ constant, resulting in

3 $$U^{*}\left( T \right) \, \sim U_{{\text{c}}} + pT{\text{ln}}\left( {t_{{\text{w}}} /t_{0} } \right),$$

where *U*_c_ for collective (elastic) pinning is lower than the characteristic pinning energy for plastic pinning (when the pinning structure accommodates vortices). Obviously, a sign changing of *p* is clearly revealed using Eq. (3), rather than the temperature variation of the normalized relaxation rate *S*(*T*) = *T*/[*U*_c_ + *pT*ln(*t*_w_/*t*_0_)]. The creep exponent can be accurately determined from magnetic relaxation measurements and Eq. (2), with the assumption that ln(*U*_c_) and ln(*J*_c0_) are weakly temperature dependent, which is true for a relatively large *T* interval at fixed DC fields^31^.

Another method of determining the pinning potential in superconducting materials is by using frequency-dependent AC susceptibility measurements as function of AC amplitude, at fixed temperatures and fixed DC fields, the method being first employed for BaZrO_3_-doped YBa_2_Cu_3_O_x_ (YBCO) superconducting thin films^32^, and later on for various YBCO films with nanoengineered pinning centers having different pinning architecture^33^. In brief, frequency-dependent critical current density was estimated from the out-of-phase (imaginary) AC susceptibility χ” response as function of the AC field amplitude *h*_ac_, at fixed temperatures *T* and fixed applied DC magnetic fields *H*_DC_. At a given temperature, for several *H*_DC_, χ”(*h*_ac_) dependence may show a peak in the experimental window of our measurements, its position h* representing the AC field of full penetration of the perturbation in the center of the sample, which can be correlated with the critical current density of the specimen^34^. In the case of our CaK1144 single crystal, the experimental window for the method to work was very close to the critical temperature, due to the exceptionally high critical current density at lower temperatures and very steep irreversibility line. Since the thickness *c* of the crystal, 0.04 mm, is much smaller than the other two dimensions, we have used the Brandt approach on the magnetic flux penetration^35^ which relates the critical current density to the position of the maximum h* in the χ”(*h*_ac_) dependence as

4 $$J_{{\text{c}}} = h^{*}/\alpha c$$

where α ≈ 0.9 is a coefficient that depends slightly on the geometry, with h* in Oe, *c* in cm, and *J*_c_ in A/cm^2^, Bradt’s original choice of units being more practical that the International System. The exact frequency (time) dependence of the critical current density (or, in the case of arbitrary geometries, of the field for full penetration h*) depends on the models of pinning involved, more precisely on the predicted dependence of the pinning potential *U* on the probing current (which is proportional to *h*_ac_). The Anderson-Kim model^26^ assumes that flux creep occurs due to thermally-activated jumps of isolated bundles of flux lines between two adjacent pinning centers, the jump being correlated for a bundle of vortices of volume *V*_c_ (correlated volume) due to the interaction between vortices, and there is a linear dependence of the pinning potential on the current density. Another well-established model is the Larkin–Ovchinnikov collective weak pinning model^23^ which considers the cooperative aspects of vortex dynamics in which the formation of the vortex lattice will be a result of a competition between the vortex-vortex interaction which tends to place a vortex on a lattice point of a periodic hexagonal/triangular lattice (Abrikosov lattice) and the vortex-pinning center interaction which tends to place a vortex on the local minimum of the pinning potential. In this model is predicted a power-law dependence of the pinning potential on the current density. In the case of various YBCO films^32,33^ a model predicting a logarithmic dependence of the pinning potential^36^ proved to be more suitable.

## Results and discussion

**Figure 1:**
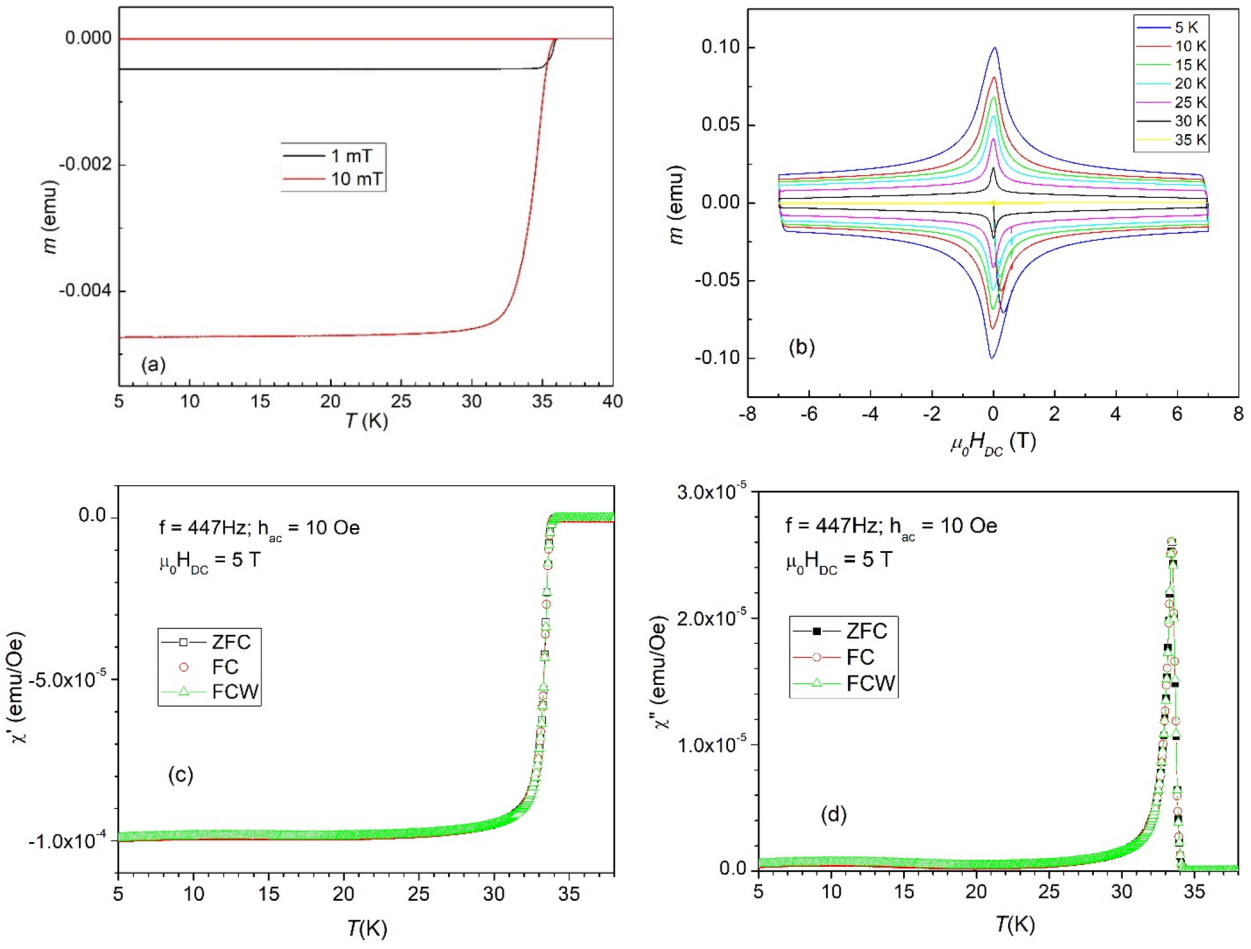
Temperature dependence of the magnetization in applied DC fields of 1 and 10 mT (**a**); magnetization hysteresis loops at several temperatures between 5 and 35 K, step 5 K (**b**); temperature dependence of the in-phase susceptibility response in a DC field of 5 T, measured with an AC field with amplitude of 10 Oe and frequency of 447 Hz, in the three cooling procedures (**c**); temperature dependence of the out-of-phase susceptibility response response in a DC field of 5 T, measured with an AC field with amplitude of 10 Oe and frequency of 447 Hz, in the three cooling procedures (**d**).

Similar CaKFe_4_As_4_ single crystals as the ones used for this study (grown by the same group in Tsukuba using the same procedure) have been thoroughly investigated using high-resolution microscopy, as well as magnetometry for estimation of critical current density^19–21^. Here we performed a limited set of measurements, presented in Fig. 1, to assess the quality of the single crystals, before embarking on the main subject of the work, namely the pinning potential.

Figure 1a shows the temperature dependence of the magnetic response, in small DC magnetic fields (1 and 10 mT), in zero-field-cooling conditions (cooling the sample in zero field, apply the magnetic field, and measure during warming-up the sample). It can be seen a very sharp superconducting transition, with a critical temperature *T*_c_ of about 35.8 K. Figure 1b presents DC magnetic hysteresis loops for DC fields between − 7 T and 7 T applied perpendicular to the *a*–*b* plane of the sample (the largest two dimensions, each about 1.2 mm), for temperatures between 5 and 35 K, as indicated in the graph. The loops look very similar to those presented in Ref.^19^ for single crystals grown using the same procedure. A complimentary analysis of the quality of the material in addition to those already presented in the literature is shown in Fig. 1c and d, where are presented the in-phase and, respectively, the out-of-phase components of the complex AC susceptibility response as function of temperature, using an AC field excitation with a frequency of 447 Hz and amplitude of 10 Oe, in a high DC applied field of 5 T. The measurements were taken using all three cooling regimes: zero-field-cooling (ZFC), field-cooling (FC) with measurement performed during cooling the sample in the applied field, and field-cooling during warming up (FCW) which means cooling the sample in the applied DC field and performing the measurement during the subsequent warming up. As can be seen in the figure, there are practically no differences between the three regimes, suggesting a very good quality superconducting single crystals with very robust superconducting properties, with absolutely no magnetic history effects, unlike many other single crystals of iron-based systems.

### Pinning potential from DC magnetic relaxation

**Figure 2:**
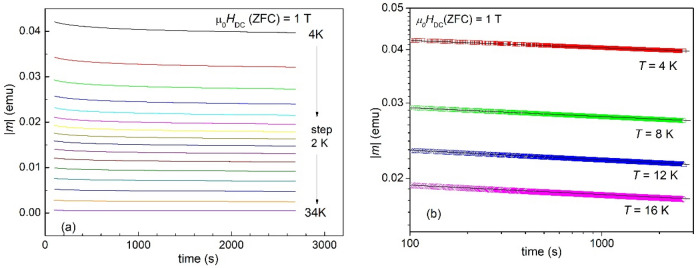
Magnetization relaxation measurements after zero-field-cooling (ZFC) in 1 T, at various temperatures between 4 and 34 K, step 2 K, excluding initial data for *t* < 100 s due to magnetic flux lines rearrangement (**a**); double logarithmic plot of selected (for clarity) magnetic relaxation data. Straight lines provide the slopes of the dln(|*m*|)/dln(*t*), averaged up to *t*_w_ = 2700 s, from which the (dimensionless) normalized relaxation rate *S* = − dln(|*m*|)/dln(*t*) can be determined (**b**).

As described in the section dealing with experimental and methods, magnetic relaxation measurements were performed after ZFC in 1, 3, and 5 T, at several fixed temperatures, for a time window *t*_w_ = 2700 s. For data analysis, first 100 s were ignored due to the well-known rearrangement of flux lines after the application of the field. Figure 2a presents an example of such magnetization relaxation measurement, for the applied field of 1 T. Figure 2b presents several examples of magnetization relaxation curves in a double-logarithmic plot. As described in the previous section, for samples with relevant pinning, if the time window is not too large (which is our case), the data in the double logarithmic plot can be very well approximated by a straight line, the absolute value of its slope being the normalized relaxation rate *S* = − dln(|*m*|)/dln(*t*), often noted in literature as S* = − Δln(|*m*|)/Δln(*t*) to stress the independence of normalized relaxation rate on time, if the time window *t*_w_ is not too large, but is large in comparison with the macroscopic time for flux creep (~ 10^−6^–1 s). In other words, we considered that in the time-frame of our measurements (*t*_1_ = 100 s < t < *t*_w_ = 2700 s), the slope is constant in time, and it can be seen that the data in Fig. 2b are indeed well described by straight lines. On the other hand, it can be clearly seen that the change in time (at a fixed temperature) of |*m*|∝ *J* is much smaller that the change due to the variation of temperature, a fact that we will use later on.

**Figure 3:**
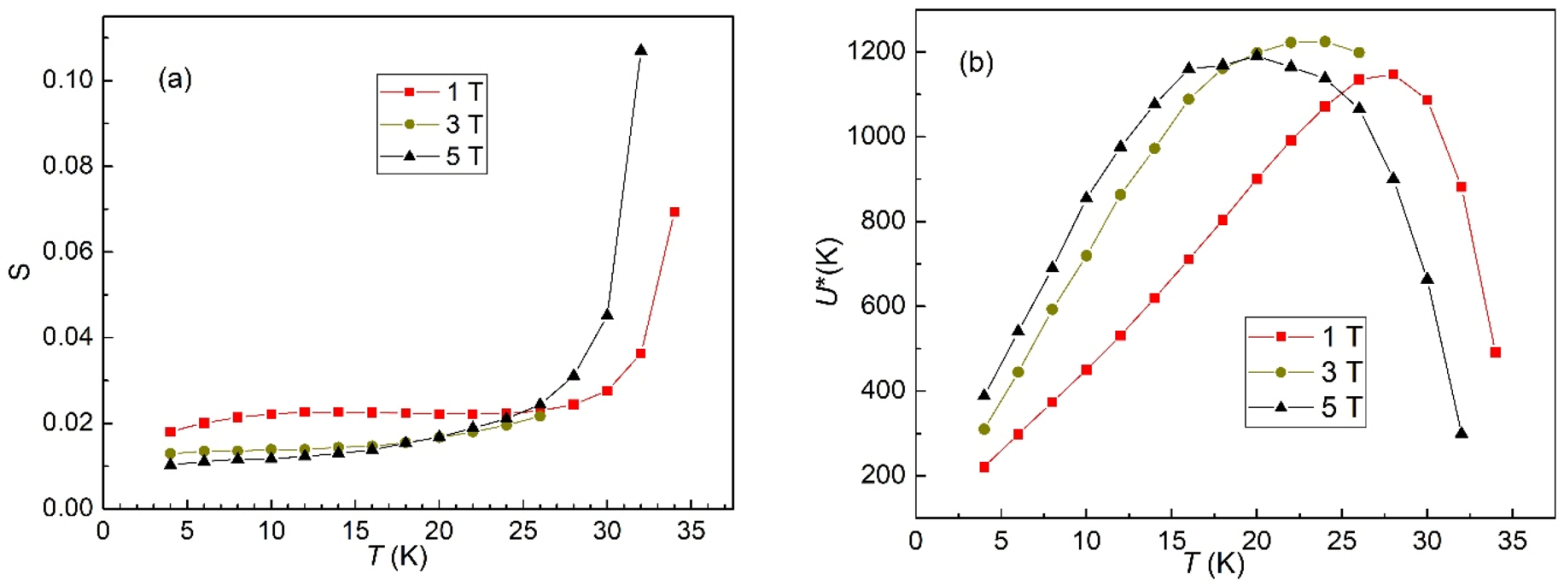
Temperature dependence of the normalized relaxation rate *S* = − dln(|*m*|)/dln(*t*) (**a**); and of the normalized pinning potential U* = *T*/*S* (**b**).

Using the procedure described in Fig. 2b we have estimated the values of *S* at various temperatures, in the three applied DC fields, which are shown in Fig. 3a, while the temperature dependence of the normalized pinning potential U* = *T*/*S* is shown in Fig. 3b. Please note that due to instrument limitation we have not been able to measure the magnetization relaxation in 3 T for temperatures higher than 27 K.

As can be seen in Fig. 3b the temperature dependence of the normalized pinning potential has a maximum that separates the elastic creep at lower temperatures from plastic creep at higher temperatures, situated between 28 K in 1 T and 20 K in 5 T, which is very important in respect with practical applications in high magnetic fields at liquid hydrogen temperatures (20 K) in the future hydrogen economy. Another important issue is the very high values of U*, up to 1200 K at peak position. Considering that the vortex creep is in principle a thermally-activated Arrhenius process, the probability of a thermally-activated flux jump (flux creep) out of the potential well associated with the pinning centers is inversely-proportional to the exponential of U*/*T* (U* in K, *k*_B_ = 1), which, for 5 T at 20 K (where U* ≈ 1200 K), is exp (− 1200/20) = exp (− 60) ≈ 9 × 10^–27^, an extremely low value. For the lower values of U* (between 200 and 400 K at lowest temperature of 4 K, liquid helium temperature, the above-mentioned probabilities are exp (− 200/4) = exp (-25) ≈ 10^–11^, and, respectively, exp (-400/4) = exp (− 100) ≈ 4 × 10^–44^. With these values, we can confidently assume that in CaK1144, at temperatures of practical interest, the dissipation due to thermally-activated flux jump (flux creep) is negligible. Even at higher temperatures, in the plastic creep regime (which, anyway, is not intended for power applications) such probabilities are small, e.g., for U* ≈ 200 K at *T* ≈ 32 K, we have exp (-200/32) = exp (− 6.25) ≈ 2 × 10^–3^.

**Figure 4:**
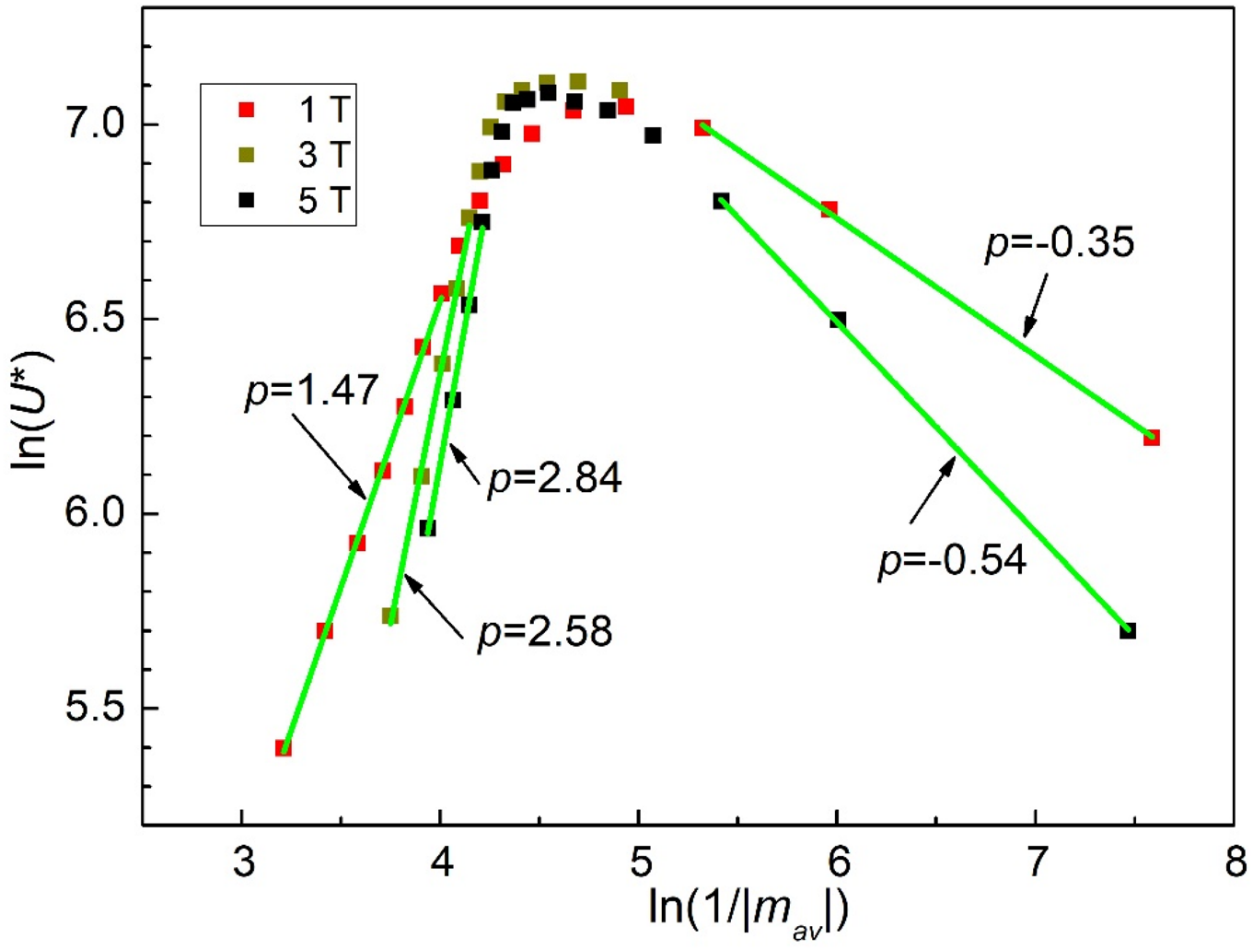
Dependence of the normalized pinning potential U* on the inverse of irreversible magnetization averaged over *t*_w_, in double logarithmic plot, for the three DC magnetic fields indicated in the figure. The slopes of the straight lines in the figure represent the vortex creep exponent *p* in Eq. (2), positive values indicating elastic creep, while negative values indicating plastic creep.

When a large current is passed through the superconductor in an applied magnetic field, Lorentz force, which is proportional to the vector product between the magnetic field and the electric current, induces flux movement. It is clear that this is the case for high-field applications, and, consequently, the dependence of the pinning potential due to variation of current (at given applied fields) is very important. Equation (2), which represents the pinning potential at fixed temperature and magnetic field as function of the current density *J* ∝|*m*|, allows the estimation of the creep exponent *p*. In our analysis we take into account the fact that, for small *t*_w_, the time dependence of |*m*| is negligible in comparison with the dependence of |*m*| on temperature, as can be seen in Fig. 2a and b. Therefore, in estimating the creep exponents, we will consider a *J*_av_(*T*,*H*) ∝|*m*_av_| (*T*,*H*) averaged over our time window, |*m*_av_|= (|*m*|_(*t*=*t*1=100 s)_ +|*m*|_(*t*=*t*w=2700 s)_)/2. Making use of the experimental data for each temperature and field and of Eq. (2) rewritten as ln(U*) = *p*[ln(1/|*m*_irr_|) + *const*], the plot ln(U*) versus ln(1/|*m*_av_|) = ln(1/*J*_av_), shown in Fig. 4, provides the needed information regarding the dependence of the normalized vortex creep activation energy (pinning potential) on the current density, in various DC magnetic fields. Please note the relationship between the experimental points in Fig. 3b, U*(*T*), and in Fig. 4, ln(U*) vs. ln(1/|*m*_av_|): for each DC field, each of the points in Fig. 4 counted from small to large ln(1/|*m*_av_|), i.e., from left to right, correspond to the temperatures in U*(*T*) plot counted from the smallest to the largest. Figure 4 also reveals a crossover between elastic creep (left-hand side of the graph) and plastic creep (right-hand side). As can be seen from Eq. (2), the slopes in Fig. 4 where ln(U*) vs ln(1/|*m*_*av*_|) dependence is linear represent the vortex creep exponent *p*, which is positive for elastic creep and negative for plastic creep (− 0.35 in 1 T, − 0.54 in 5 T, in good agreement with theoretical predictions *p* =  − 0.5 for dislocation-mediated plastic creep).

The values of the vortex creep exponents in the elastic creep regime are also in striking agreement with theoretical predictions: in 1 T *p* = 1.47 is practically 3/2 as predicted by the elastic creep of small vortex bundles, while in higher fields, 3 and 5 T, *p* values of 2.58 and, respectively, 2.84 are comparable with the theoretical prediction from the elastic manifold theory in the small vortex bundle creep regime *p* = 5/2.

### Pinning potential from AC susceptibility

**Figure 5:**
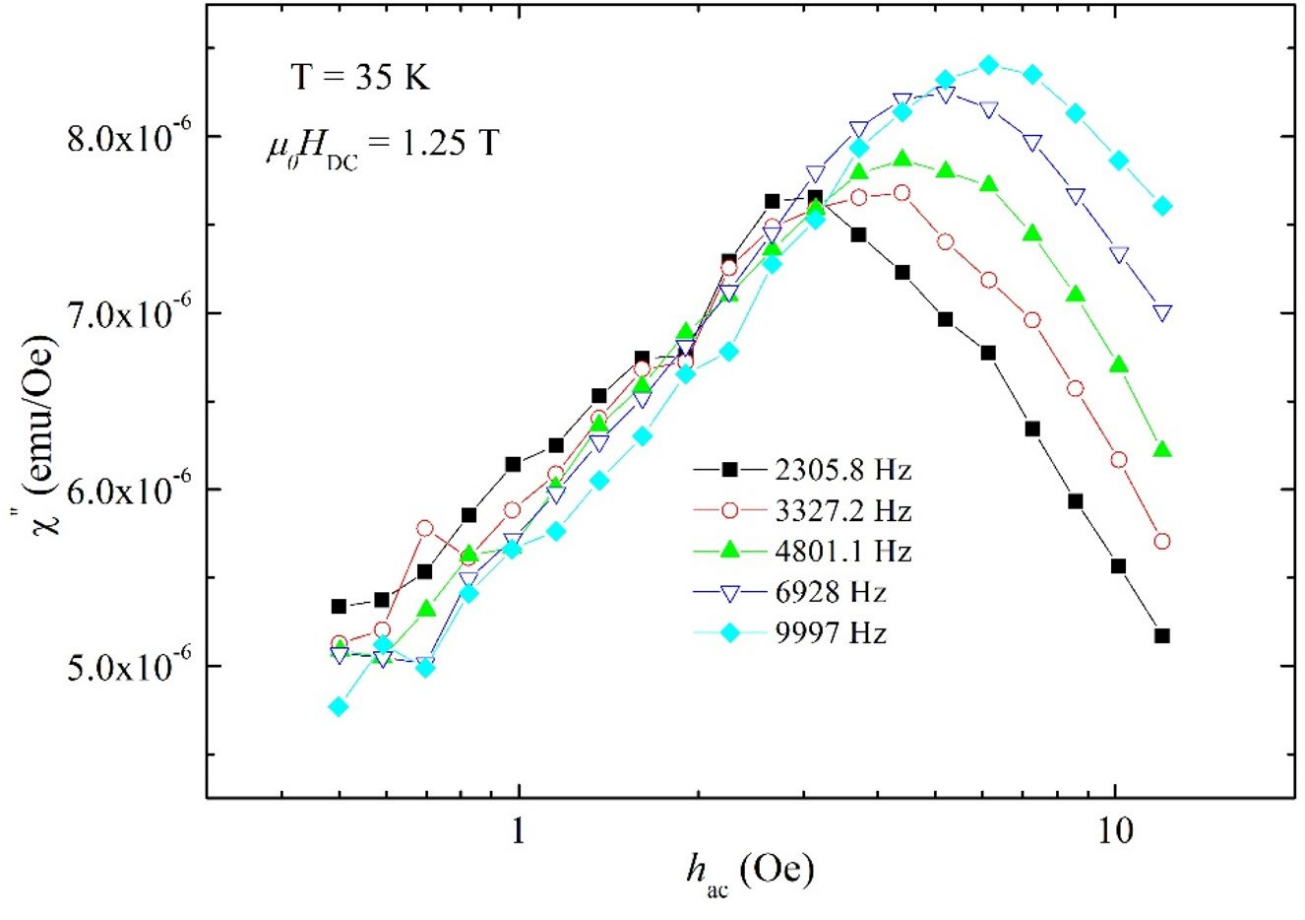
Dependence of the out-of-phase susceptibility on the AC field amplitude, at 35 K and in DC field of 1.25 T, for various AC field frequencies, as indicated in the figure.

As briefly described in the experimental and method section, another technique of estimating the pinning potential, applicable in the case of thin superconducting samples with fields perpendicular to the largest area, implies setting constant temperatures and DC magnetic fields, and measuring the dependence of the out-of-phase susceptibility as function of the amplitude of the AC excitation field, for several frequencies of the AC field. One example of such measurement is presented in Fig. 5, for a fixed temperature of 35 K, DC field 1.25 T, and AC frequencies indicated in the figure. As mentioned in the method section, due to the very large critical current density in CaK1144, and to the equipment limitation regarding the maximum available *h*_ac_ (16 Oe), with this method we had to probe a region in which critical current density is small enough to ensure that the corresponding h* (see Eq. (4)) will not exceed 16 Oe, so we could obtain the desired results only very close to the critical temperature, in the plastic creep regime. As can be seen in Fig. 5, the position of the maximum (h*) in the χ”(*h*_ac_) dependence shifts towards higher values with increasing frequency.

**Figure 6:**
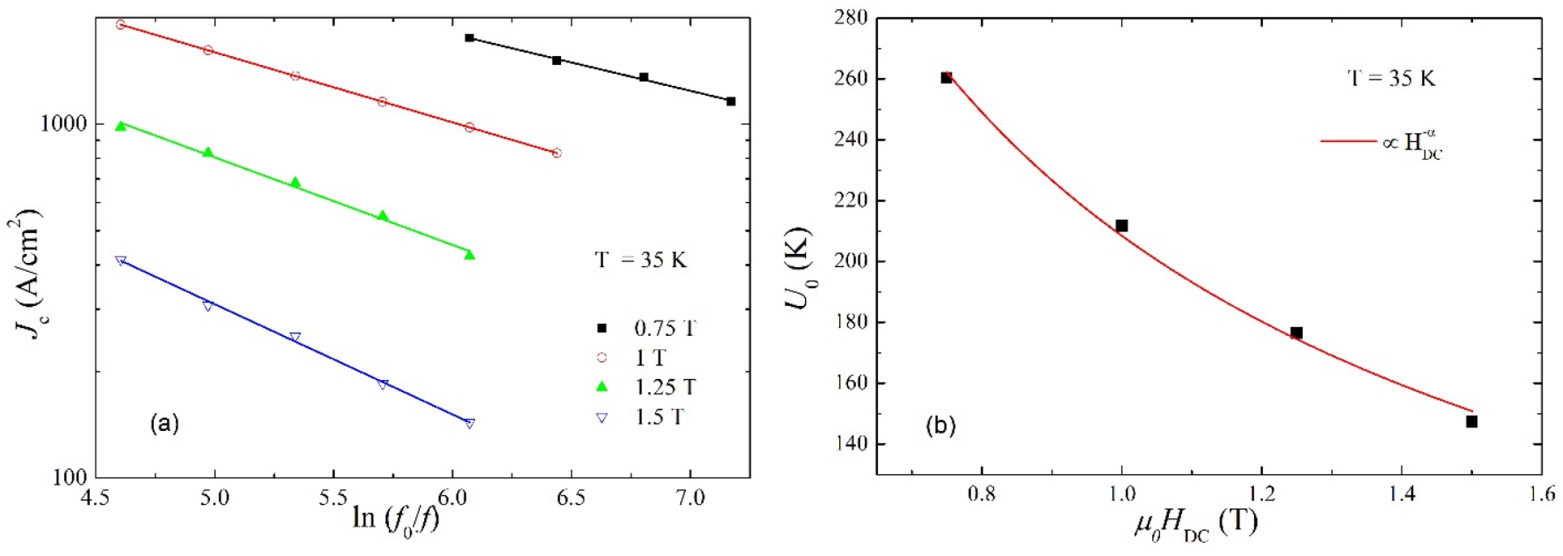
Frequency dependence of the critical current density, calculated from the susceptibility measurements using Brand’s formula, at 35 K, in the DC fields indicated in the figure, in double-logarithmic plot. The slopes *b* of the linear fits allows to estimate the values of the pinning potential *U*_0_ (**a**); DC field dependence of the pinning potential, in K (*k*_B_ = 1). Full line represent a power-law fit ${U}_{0}\propto {H}_{DC}^{-{\alpha}}$, with α ≈ 0.8 resulting from the fitting procedure (**b**).

By using Eq. (4), from the h* we estimated the critical current densities at various frequencies (where a maximum in χ”(*h*_ac_) could be found), at 35 K, and in several DC applied fields. Figure 6a shows the dependence of *J*_c_ (in logarithmic scale) on ln(*f*_0_/*f*), where *f*_0_ is a macroscopic attempt frequency of about 10^6^ Hz. The shape of the experimental dependence *J*_c_ (in logarithmic scale) vs ln(*f*_0_/*f*) gives indication on the suitability of various models of pinning^33^: a downward curvature indicates an Anderson-Kim model of pinning^26^, an upward curvature indicates collective pinning model^23^, while a straight line (as is the case of our data) indicates the suitability of a logarithmic dependence of the pinning potential on the current density^36^. As was demonstrated in^33^, for the latter case, the slope *b* of the straight lines in Fig. 6a allows the estimation of pinning potentials for each DC applied field, namely *U*_0_ = *k*_B_*T*(1 + 1/*b*). The resulted values of *U*_0_, in K (*k*_B_ = 1) are shown in Fig. 6b, for each DC field.

Although there are only four experimental points, the *U*_0_ vs *H*_DC_ behavior can be fitted by the power law ${U}_{0}({H}_{DC})\propto {H}_{DC}^{-\alpha }$, where *α* ≈ 0.80, consistent with both collective pinning and Zeldov model due to the large number of vortices present in the sample at given temperature and DC fields.

Comparing the two methods, we have estimated the normalized pinning potential from DC magnetization relaxation measurements in a large temperature range, between 4 K and critical temperature, sweeping elastic creep, plastic creep, and the crossover between the two regimes, while in the case of frequency-dependent AC susceptibility measurements, the very high critical current density of this superconducting material allowed us to probe the vortex dynamics only in the plastic creep regime, very close to the critical temperature. However, extrapolating U*(*T*) dependence from magnetization relaxation in Fig. 3b for *H*_DC_ = 1 T towards *T* = 35 K, one can estimate U*(35 K, 1 T) to be between 200 and 250 K, and a closer look at Fig. 6b will show a value from AC susceptibility at the same temperature and DC field, *U*_0_ (35 K, 1 T) ≈ 210 K, which is quite striking considering the much different timescale of the two types of measurements, 10^3^ s, and, respectively, 10^–3^ s. The most likely explanation of this fact is that, very close to *T*_c_, the viscosity of the plastic vortex matter is very small (practically becomes a vortex liquid), and the timescale of the low viscosity plastic creep is less important.

In conclusion, we have investigated high-quality single crystals of the highly performant superconducting material CaKFe_4_As_4_ using isothermal DC magnetization, DC magnetization relaxation and frequency-dependent AC susceptibility. Critical temperature, the shape of the superconducting transition, and the shape of isothermal magnetization loops are similar to those previously reported in the literature on similar single crystals grown in the same laboratory. Temperature dependence of the AC susceptibility in high DC magnetic fields revealed absolutely no difference due to the magnetic history, measurements done in zero-field-cooling, field-cooling, and, respectively, field-cooling and warming up give the same results. From magnetization relaxation measurements were extracted the values of the normalized pinning potential U*, and its temperature dependence show a clear crossover between elastic creep and plastic creep. The extremely high values of U*, up to 1200 K around the temperature of 20 K lead to a nearly zero value of the probability of thermally-activated flux jumps (flux creep) at temperatures of interest for high-field applications. The values of the creep exponents in the two creep regimes were also estimated, their values being in complete agreement with theoretical models. Using frequency-dependent AC susceptibility at a fixed temperature (close to the critical temperature) and DC fields we have estimated the AC excitation field of full penetration, the frequency-dependent critical current density and the field-dependent pinning potential *U*_0_. To our surprise, the value of U* (35 K, 1 T) determined from magnetization relaxation is very close to the value of *U*_0_ (35 K, 1 T) determined by the AC susceptibility technique.

## Data Availability

The data sets that support the findings in this study are available from the corresponding author upon reasonable request.
